# Pulmonary artery intimal sarcoma: Case report of a patient managed with multimodality treatment and a comprehensive literature review

**DOI:** 10.1007/s00066-024-02250-6

**Published:** 2024-06-12

**Authors:** C. Atahan, Z. Güral, S. Yücel, F. Ağaoğlu

**Affiliations:** Department of Radiation Oncology, Acibadem Mehmet Ali Aydinlar University School of Medicine, Istanbul, Turkey

**Keywords:** Vascular tumor, Radiation, Surgical resection, Adjuvant chemotherapy, Radiotherapy

## Abstract

Pulmonary artery intimal sarcoma (PAIS) is a rare and aggressive malignancy originating from the intimal layer of the pulmonary artery with poor prognosis due to its aggressive nature. The management of PAIS poses both diagnostic and therapeutic challenges. It presents with nonspecific symptoms and is often misdiagnosed as pulmonary embolism. While surgical resection is the primary treatment modality, the role of adjuvant chemotherapy and radiotherapy remains uncertain. However, given the high recurrence rate, adjuvant chemotherapy and/or radiotherapy have been utilized in a limited number of case reports. We present the case of a 46-year-old woman who was diagnosed with PAIS and underwent surgical resection followed by adjuvant chemotherapy (ChT) and radiotherapy (RT), demonstrating good tolerance to this multimodal treatment approach.

## Introduction

Pulmonary artery sarcoma is a rare malignancy with poor prognosis and presents challenges in diagnosis and treatment. While surgical resection is the primary treatment modality, some studies have shown that ChT or RT can be effective against pulmonary artery sarcoma, but the overall prognosis is exceptionally poor. In the context of RT specifically, there is limited documented experience with its use in pulmonary artery sarcoma. Thus, we present a case of PAIS patient who underwent surgery and received adjuvant ChT and RT, along with a review of the literature.

## Case presentation

**Fig. 1:**
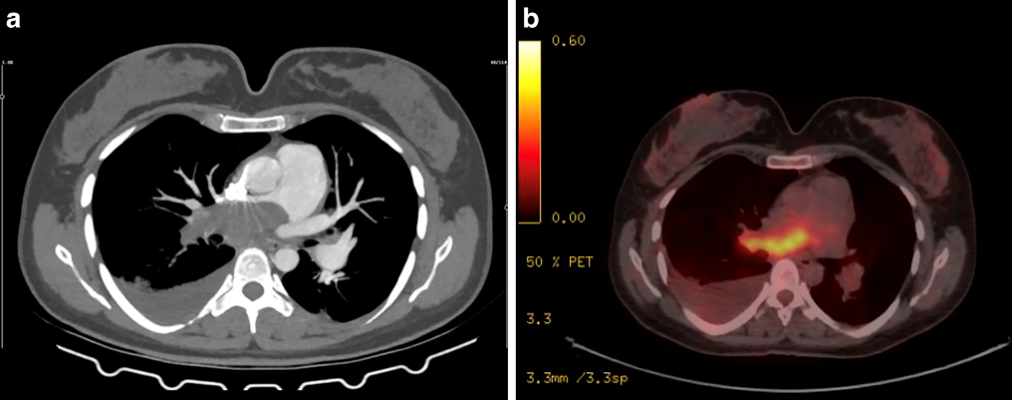
Axial computed tomographic (CT) pulmonary angiogram and 18F-fluorodeoxyglucose positron emission CT (FDG PET-CT) scans at the initial presentation. **a** CT pulmonary angiogram shows a large filling defect in the right main pulmonary artery extending to the subsegmental branches. **b** FDG PET-CT shows high metabolic activity in the right pulmonary artery consistent with CT pulmonary angiogram findings

A 46-year-old woman presented to the emergency department with a 3-month history of progressively worsening dyspnea, cough, and thoracic discomfort encompassing the chest and back. Her medical history was unremarkable, with no known pathologies, and she was a nonsmoker. Chest computed tomography (CT) demonstrated the presence of a lesion with a 3 cm diameter in the right pulmonary artery, causing near total occlusion of the artery. CT pulmonary angiogram revealed a substantial occlusive filling defect spanning the pulmonary trunk and extending into the right main pulmonary artery and segmental arteries, suggestive of pulmonary embolism with potential focal pulmonary infarction (Fig. 1a) and pleural effusion in the right hemithorax. D‑dimer level was 849 ng/ml (normal range 0–500 ng/ml) and CRP level was 67 mg/L (normal range 0–5 mg/L). The results of complete blood counts, hepatic and renal function tests, serum electrolyte analysis, cardiac biomarker assessment, autoantibody screening, and coagulation studies were within normal limits. Doppler ultrasound of lower limb and carotid and vertebral arteries was found to be normal. Echocardiography revealed normal ventricular functions and mild tricuspid insufficiency with a pulmonary artery pressure of 25 mm Hg. Subsequently, the patient was diagnosed with pulmonary embolism and anticoagulation therapy was initiated with a 4-week course of low-molecular-weight heparin. Due to unresponsiveness to the treatment, further investigation was needed. 18F-fluorodeoxyglucose positron emission computed tomography (FDG PET-CT) imaging for tissue characterization of the primary pulmonary artery mass revealed elevated FDG uptake SUVmax 10 (maximum standardized uptake value) within the lesion of the right pulmonary artery (Fig. 1b).

The patient underwent pulmonary endarterectomy involving the main and bilateral pulmonary arteries, under cardiopulmonary bypass. Histological examination revealed proliferation of spindle cells characterized by pleomorphic nuclei. Immunohistochemical staining demonstrated focal positivity for SMA and desmin, whereas pansitokeratin, S100, and CD34 were negative. the proliferation index Ki-67 was recorded at 80%. Fluorescence in situ hybridization (FISH) test confirmed the diagnosis of PAIS by detecting mouse double minute 2 (MDM2) gene amplification. Subsequent next generation sequencing (NGS) analysis revealed a TFG::ADGRG7 fusion, classified as Tier III, which was interpreted as clinically insignificant. The patient’s postoperative course was uncomplicated. Following surgical treatment, the patient exhibited significant symptomatic improvement with resolution of dyspnea and chest pain.

Patient was evaluated by our multidisciplinary tumor board, leading to the decision for adjuvant therapy comprising sequential ChT and RT. One month after surgery, the patient commenced a ChT regimen consisting of doxorubicin at a dosage of 60 mg/m^2^ (on day 1), ifosfamide at 1800 mg/m^2^ (on days 1, 2, 3, 4, 5) and mesna (2-mercaptoethane sulfonate Na [sodium]) at 2000 mg/m^2^ (on days 1, 2, 3) administered every 21 days for a total of six cycles over 4 months. She tolerated ChT very well without any severe acute toxicity.

**Fig. 2:**
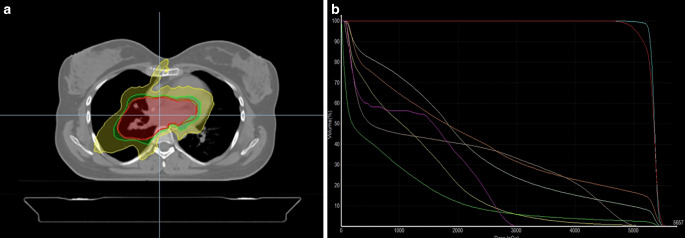
Radiation therapy plan of the presented case. **a** Computed tomography-based plans of intensity-modulated radiotherapy showing isodose levels in color washes. *Red*, *green*, and *yellow* colors encompass the areas covered by 95%, 80% and 50% of the prescribed dose, respectively. **b** Dose–volume histogram of the treatment plan. *Green*, *yellow*, *light pink*, *light green*, *magenta*, *orange*, *red*, and *turquoise* *lines* presents total body, left lung, right lung, esophagus, spinal cord, heart, planned target volume (PTV) and gross tumor volume (GTV), respectively

An FDG PET-CT scan conducted 1 month postcompletion of ChT and 7 months postsurgery revealed no pathological FDG uptake. Subsequently, adjuvant RT targeting the tumor bed was planned using helical tomotherapy based (Accuray, Sunnyvale, CA, USA) intensity-modulated RT with daily image guidance, administering a total dose of 60 Gy in 30 fractions, delivered over five fractions per week (Fig. 2). She demonstrated excellent tolerance to RT with only mild dysphagia as a side effect.

## Discussion

PAIS is an extremely rare tumor with a probably underestimated incidence of 0.001–0.03% [1–3] due to challenges in diagnosis. It mainly occurs in middle-aged adults with a broad spectrum of ages of onset between 13 and 86 [4]. It progresses aggressively if left untreated. The median survival rates reported in the series are approximately 1.5 months without treatment [5] and 17 months with intervention [6].

PAIS symptoms are usually nonspecific, encompassing cough, exertional dyspnea, chest pain, and signs of right ventricular failure. The symptoms and radiological imaging findings can often resemble those of pulmonary embolism, potentially leading to a delayed diagnosis. However, reports indicate that symptom onset in PAIS typically progresses more gradually than in pulmonary embolism [7]. FDG PET-CT imaging may prove valuable in distinguishing PAIS from pulmonary embolism, utilizing the SUVmax parameter. PAIS typically exhibits a higher SUVmax compared to pulmonary thromboembolism, with a specificity of 97% when the SUVmax exceeds 3.3 [8]. Nevertheless, one should be cautious as some tumors might show a low FDG uptake [3]. Definitive diagnosis is made by pathological examination of the tissue, which is usually obtained during surgery. Intimal sarcoma arises from intimal layer of large blood vessels, such as the pulmonary arteries and aorta. Intimal sarcomas of the pulmonary circulation are mostly observed in the pulmonary trunk or in the main right and/or left pulmonary artery [3]. Histologically, leiomyosarcoma (21%) and spindle cell sarcoma (15%) are the most common subtypes, whereas liposarcoma is the least common (1%) [7, 8]. As intimal sarcoma of the pulmonary artery is seen more frequently than intramural pulmonary artery sarcoma, ‘pulmonary artery sarcoma’ is often referred to as PAIS.

Due to it being a rare tumor, an optimal treatment strategy has not been established; nevertheless, surgery is the backbone of treatment, including pulmonary endarterectomy, lobectomy, or pneumonectomy. Initially, pneumonectomy through thoracotomy was considered the gold standard for treating these tumors. However, due to the tumors’ proximal origin and centrifugal extension from the pulmonary artery intima, as well as their invasion of surrounding structures, it became evident that radical resection via sternotomy under cardiopulmonary bypass is necessary. This approach minimizes the risk of leaving any residual tissue, thereby potentially improving patient outcomes. Pulmonary endarterectomy under deep hypothermia and circulatory arrest is preferred whenever possible, as it offers the potential for complete tumor removal [1].

The role of adjuvant therapy is not clear, although there are reports of cases with improved prognosis with the use of ChT and/or RT [5, 6, 9, 10]. A review from the MD Anderson Cancer Center found a median survival of 25 months for patients undergoing multimodal treatment compared with 8 months for patients who received single-modality therapy [10]. If adjuvant ChT is used, there is no consensus regarding which agent should be administered. Most ChT regimens reported in the literature contain anthracyclines. However, PAIS is highly resistant to ChT, and disease progression usually occurs within months of ChT completion [3]. Although adjuvant ChT improved survival and distant metastasis in one analysis, it had no effect on local recurrence [7]. However, the role of adjuvant RT remains unclear. Prognosis is significantly worse in patients who undergo partial resection compared to those of with complete resection [7, 10] and deaths are usually due to recurrence in distal pulmonary arteries, local recurrence, and metastasis [11]. Thus, adjuvant RT administration has been reported in several cases [1, 3, 5, 6, 9, 12]. In a case series in which 2 of 6 patients had adjuvant RT with concurrent ChT, the authors reported that patients who received adjuvant therapy had better survival [9], while in another case series involving 31 patients, where the adjuvant therapy group consisted of 2 patients with only RT, 15 patients with only ChT and 1 patient with both treatments, no significant statistical difference in survival was found in the adjuvant therapy group [1]. Wong et al. found adjuvant therapy improved median survival (24 months vs 8 months) in their series where 4 and 5 patients of 20 had received RT and ChT, respectively. They also reported that the longest surviving patient (102 months) had undergone surgery, adjuvant ChT, and RT [6]. A Korean study of 11 of 20 patients who received adjuvant treatment (ChT = 3, RT = 5, chemoradiotherapy = 3) indicated that adjuvant ChT was associated with improved survival [12]; in a case series of 13 patients from Leuven University Hospital, 2 patients underwent postoperative RT and no adjuvant ChT was administered to any patient, and they reported no evidence of disease after finishing RT, but both developed metastatic disease during follow-up. RT doses in these studies ranged between 50 and 66 Gy with conventional fractionations [3, 5]. Since local recurrence was reported in nearly half of cases after resection, and distant metastasis was reported in approximately 20–30% [13–15], we considered adjuvant treatment with ChT and RT necessary in the management of our patient even though she underwent complete resection of the tumor. Our patient has not experienced any complications or severe side effects during her treatment. However, treatment tolerance of the patient throughout surgery, ChT and RT might be a concern for the treating physician. Some series reported perioperative mortality between 13% [1] and 20% [6], while others reported no mortality due to surgery [5]. Overall ChT and RT toleration was reported as either well or was not mentioned at all in the literature [1, 5, 6].

In selected cases, use of neoadjuvant therapy prior to surgery or definitive ChT or ChT and RT without surgery has been reported [1, 15–18]. Neoadjuvant RT was reported in two case reports and might be considered to improve resectability by shrinking tumor [15, 16]. However, neoadjuvant ChT was not found to be useful in terms of tumor shrinkage prior to surgery [3]. One patient who had been successfully treated with ChT (two cycles of doxorubicin, cisplatin, and ifosfamide and four cycles of vinorelbine and cisplatin) without surgery was also reported. The definitive use of ChT and RT without surgery was also reported in three cases. One patient with recurrent disease who had not received adjuvant treatment previously received two courses of carboplatin ChT with subsequent 53 Gy RT. Although they reported 60% tumor shrinkage, metastases were observed 7 months after RT [17]. In another recurrent disease case who had been treated previously with both adjuvant ChT and RT, the patient had 4 cycles of docetaxel and gemcitabine followed by 24 Gy RT in 4 fractions. The tumor diameter was reduced from 3 to 2 cm after reirradiation [19]. The other patient received concurrent paclitaxel ChT and 66 Gy RT without surgery due to medical comorbidities. The patient continued ChT afterwards. Even when the tumor shrank very slightly on radiological examinations, the patient’s clinical symptoms improved, and he was alive 36 months after diagnosis [18]. However, the impact of such treatments is controversial because the data come from limited case reports and there is a lack of prospective randomized trials.

Experience regarding the use of molecular-targeted agents for treating intimal sarcoma is limited. It is reported that in molecular analysis most frequent gene amplifications are MDM2 (65%), cyclin-dependent kinase 4 (CDK-4), platelet-derived growth factor receptor α (PDGFRA; 81%) and epidermal growth factor receptor (EGFR; 76%) gene amplifications [3]. In the Leuven series, a single-agent tyrosine kinase inhibitor, imatinib, was administered as a first- or second-line treatment in 4 patients. The authors reported that only 1 patient achieved a partial metabolic response on FDG PET-CT and disease stabilization for 9 months. Imatinib was poorly tolerated by 2 of the 4 patients and was discontinued. They hypothesized that owing to the clonal heterogeneity of the tumor and the potential contribution of PDGFRA and EGFR signaling pathways, imatinib did not provide disease control and concluded that the therapeutic activity of this targeted agent in PAIS cannot be determined based on their findings [3]. In a report of a metastatic patient with genetic profiling showing alterations in the DNA repair pathway, olaparib achieved transient disease control for 2 months [20].

Our patient’s FISH analysis was positive for MDM2 gene amplification, in concordance with the literature [3, 4, 21]. Targeting MDM2, either alone or in combination with other agents, could be a therapeutic approach, although MDM2 inhibitors are still undergoing clinical trials and are not yet clinically available [22]. The patient’s NGS analyses revealed a TFG::ADGRG7 fusion, which is considered Tier III as clinically insignificant and has been reported in various cancer types as well as in healthy tissue in the literature [23]. Although genomic profiling is increasingly being used in the assessment and management of sarcomas, little is known about its utilization in PAIS.

## Conclusion

Pulmonary artery intimal sarcoma (PAIS) poses a diagnostic challenge due to its nonspecific symptoms and rarity. While imaging techniques like FDG PET-CT might be beneficial for differentiation, definitive diagnosis relies on pathological examination during surgery. Despite the lack of a standardized treatment approach, multimodal therapy combining surgery, chemotherapy, and radiotherapy might show promise in improving survival. Additionally, emerging molecular targeted therapies offer potential benefits, although further research is needed. Collaboration among multidisciplinary teams and ongoing research efforts are crucial for refining treatment strategies and enhancing outcomes for patients with this challenging malignancy. We hope that our case treated with multimodality therapy contributes to the management of PAIS, as data in the literature on this rare tumor is limited to only case series.
